# Supplementary material for: Proteogenomic network analysis reveals dysregulated mechanisms and potential mediators in Parkinson’s disease
Source: Nat Commun. 2024 Jul 31;15:6430. doi: 10.1038/s41467-024-50718-x (PMC11289099; doi:10.1038/s41467-024-50718-x)
Supplement: Supplementary file 1 — Supplementary Information [file 41467_2024_50718_MOESM1_ESM.pdf]

Supplementary Methods

GWAS of clinically defined PD risk in UKB

Using UKB clinical and imputed array data, we created a cohort of 2,864 clinically defined PD patients and 158,876 controls of European (EUR) ancestry and conducted GWAS using REGENIEv2.2.4 [1] (Methods, Supplementary Data 1). A list of top identified significant associations are provided in Figure 2a and Table 1. This included 1042 female and 1822 male patients, with a median age at onset of disease of 71 years (Supplementary Data 1). Seven genome-wide significant loci (P-value threshold= 5×10^{-8}) were identified including: rs35749011-A, rs1474055-T, rs356203-T, rs34637584-A, rs7155501-G, rs56214516-C, and rs429358-C (Figure 2a and Table 1). Two of these SNPs, rs34637584 and rs429358, are missense variants for the well-established PD and AD genes *LRRK2* and *APOE*, respectively, with a Combined Annotation Dependent Depletion (CADD) score of 28.2 and 16.65 indicating that they are among the top 1% and 10% of the most deleterious genetic alterations in the genome. Both variants are annotated as pathogenic or likely pathogenic in ClinVar [2]. For each of the variants indicated in Table 1, we have included three measures including CADD [3], Regulome Database (RBD) [4], and Variant to Gene (V2G) [5] scores. RBD score is designed to guide interpretation of regulatory variants in the genome through combining a series of experimental datasets as well as computational predictions with the ultimate goal of identifying functional variants [4]. Variant to Gene (V2G) scores, is designed to provide an aggregated score to help rank likely genes for a given variant. This score is generated through combining four different data types including molecular quantitative trait loci (e.g., eQTLs), *in silico* functional prediction data, chromatin interactions, and the distance of the variant to the canonical transcription start sites [5].

Meta-analysis of UKB and FinnGen case-control GWAS

We next conducted a meta-analysis (Methods) of the UKB-curated cohort and FinnGen R9. Combining the FinnGen PD cohort of 4,235 cases and 373,042 controls led to a total number of 7,099 PD cases and 531,918 controls. In total, this meta-analysis identified 10 independent significant loci (Table 2 and Figure 2b), including three missense variants at *GBA*, *TMEM175*¸and *LRRK2* with CADD scores of 16.14, 12.9 and 28.2, respectively. We further examined the distribution of the variant effect of the significant SNPs (Figure 2c). Intronic variants accounted for 54% of all significant variants, followed by intronic non-coding RNA (27%) and intergenic variants (11.1%).

We investigated the added value of meta-analysis GWAS compared to the GWAS performed on the UKB cohort only. Upon meta-analysis, the total number of significant loci increased from 7 to 10 with some differences in the top significant SNPs. In chromosome 1, rs35749011, an intergenic variant to *KRTCAP2* was initially identified as the most significant SNP. Upon meta-analysis, rs2230288, a missense variant in *GBA* previously reported in the literature, was replicated as the most significant SNP in this chromosome. These two SNPs are in high linkage disequilibrium (LD) in the European population. In chromosome 2, *STK39* was shared in both analyses as the significant hit. In the UKB GWAS, *SNCA* was the top identified locus in chromosome 4, however upon meta-analysis, *TMEM175* as a well-known PD rick locus was also replicated alongside *SNCA*. UKB GWAS was not powerful enough to identify any significant hits in chromosome 6 while there are a few loci reported in this chromosome in the literature. Upon meta-analysis, we were able to identify several significant SNPs in which *HLA-DRB1* was

the most significant locus in this chromosome. In chromosome 12, *HIP1R* was an added significant locus upon running meta-analysis while *LRRK2* was identified in both analyses. While *GCH1* was identified in both sets of analyses in chromosome 14, the top significant hit in chromosome 17 switched from *CRHR1* to *KANSL1*. Both these loci have been reported in the literature [6]. We should note that still rs56214516 (intronic to *CRHR1*) was significant, however, since it is in high LD with rs4630591 (intronic to *KANSL1*), it was not presented as the top significant locus in this chromosome in the meta-analysis. In the UKB GWAS, rs429358 (a missense variant in *APOE*) was the only SNP significantly associated with the PD risk. Upon meta-analysis, while this SNP remained genome-wide significant, rs6857 (a 3'-UTR SNP to *NECTIN2*) presented the highest significance. These two SNPs in chromosome 19 are in high LD in the European population, therefore we only reported *NECTIN2* as the top locus in the meta-analysis results.

Supplementary Figures

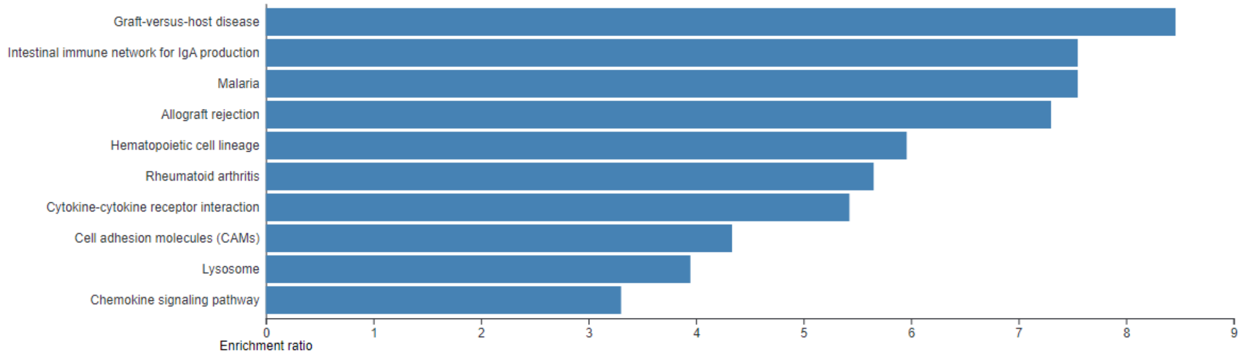

Supplementary Figure 1. The top 10 significantly enriched pathways using the 577 identified PD-associated proteins.

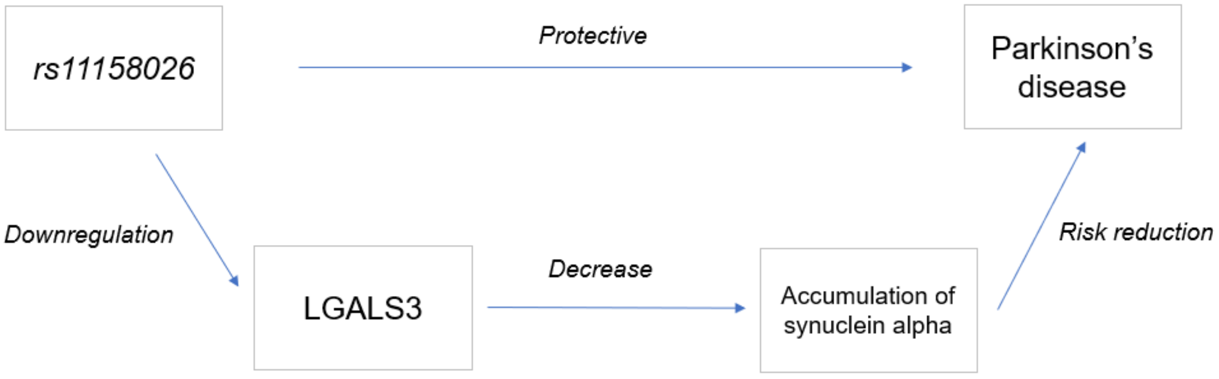

Supplementary Figure 2. The derived hypothesis on how the protective variants plays a protective role against PD.

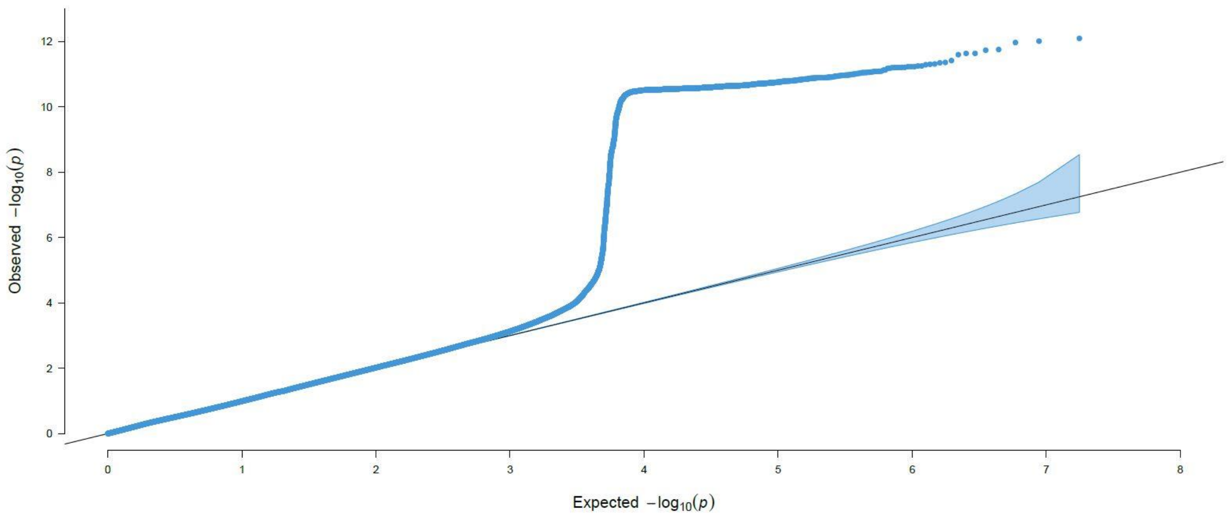

Supplementary Figure 3. The QQ plot for the UKB-FinnGen meta-analysis

Supplementary References

1. Mbatchou, J. *et al.* Computationally efficient whole-genome regression for quantitative and binary traits. *Nat Genet* 53, 1097-1103 (2021).

2. Landrum, M.J. *et al.* ClinVar: improving access to variant interpretations and supporting evidence. *Nucleic Acids Res* 46, D1062-D1067 (2018).

3. Rentzsch, P., Witten, D., Cooper, G.M., Shendure, J. & Kircher, M. CADD: predicting the deleteriousness of variants throughout the human genome. *Nucleic Acids Res* 47, D886-D894 (2019).

4. Boyle, A.P. *et al.* Annotation of functional variation in personal genomes using RegulomeDB. *Genome Res* 22, 1790-7 (2012).

5. Carvalho-Silva, D. *et al.* Open Targets Platform: new developments and updates two years on. *Nucleic Acids Res* 47, D1056-D1065 (2019).

6. Nalls, M.A. *et al.* Identification of novel risk loci, causal insights, and heritable risk for Parkinson's disease: a meta-analysis of genome-wide association studies. *Lancet Neurol* 18, 1091-1102 (2019).
