# Supplementary material for: Proteogenomic network analysis reveals dysregulated mechanisms and potential mediators in Parkinson’s disease
Source: Nat Commun. 2024 Jul 31;15:6430. doi: 10.1038/s41467-024-50718-x (PMC11289099; doi:10.1038/s41467-024-50718-x)
Supplement: Supplementary file 3 — Description of additional supplementary files [file 41467_2024_50718_MOESM3_ESM.pdf]

Description of Additional Supplementary Files

File Name: Supplementary Data 1

Description: Demographics of the study, PD-associated pQTLs, and CSF-plasma comparison analytical results.

File Name: Supplementary Data 2

Description: List of the generated gene-based scores.

File Name: Supplementary Data 3

Description: Pathway enrichment analysis.

File Name: Supplementary Data 4

Description: The identified proteins in the top 1% of PD-associated modules.

File Name: Supplementary Data 5

Description: The list of the PD variants overlapping regulatory regions
